# Children's Deformities

**Published:** 1890-06

**Authors:** 


*Children's Deformities.* By WALTER PYE, F.R.C.S. Pp. 216. Bristol: WRIGHT & Co. No date. (Preface, 1889.)

The first thing one does in looking over a book is to read the preface, and then look at the table of contents and the index. We happened, at the moment, to be interested in hypospadias, and we looked for it—it does not appear: then we sought epispadias and extroversion of the bladder—these also are absent. Then we turned to the contents, and after a perplexing study of a most heterogeneous arrangement of subjects, we read the book; and we have come to the conclusion that it is a very ordinary one, neither good nor bad, but—meriting the third epithet.

A man may be a very good practical surgeon, doing work up to the highest standard; but before he writes a book aiming at the instruction of others, he ought to read *at first hand* the original essays of the authors he quotes. And his selection of methods ought not to be merely casual, but should always have reference to accredited authority, even if it is not the author's. The author has done better work than this.

